# Combined Microsurgical and Endovascular Intracranial Aneurysm Treatment: Interdisciplinary Experience Using a True Hybrid Approach and a Systematic Review of the Literature

**DOI:** 10.3390/brainsci14080816

**Published:** 2024-08-15

**Authors:** Sabrina Ulmer, Philipp Gruber, Gerrit A. Schubert, Luca Remonda, Serge Marbacher, Basil E. Grüter

**Affiliations:** 1 Department of Neurosurgery, Kantonsspital Aarau, 5001 Aarau, Switzerland; sabrina.ulmer@ksa.ch (S.U.); gerrit.schubert@ksa.ch (G.A.S.);; 2 Division of Neuroradiology, Department of Radiology, Kantonsspital Aarau, 5001 Aarau, Switzerland; 3 Department of Neurosurgery, Universitätsmedizin Aachen, RWTH Aachen University, 52074 Aachen, Germany; 4 Division of Neuroradiology, Kantonsspital Aarau, University of Bern, 3008 Bern, Switzerland; luca.remonda@bluewin.ch; 5 Faculty of Medicine, University of Bern, 3008 Bern, Switzerland

**Keywords:** true hybrid approach, intracranial aneurysm, combined treatment, endovascular and surgical combined approach

## Abstract

(1) Background: Most intracranial aneurysms (IAs) can be treated either with microsurgical clipping or endovascular techniques. In a few cases, simultaneous treatment utilizing both modalities in a hybrid operation room may be favorable. This study analyzes the indication and benefits of a true hybrid approach (tHA) that combines simultaneous endovascular and microsurgical procedures for treatment of IAs in one session. (2) Methods: All patients receiving a true hybrid procedure between 2010 and 2022 in our institution were included. Demographic characteristics, neurological symptoms, pre-interventional treatments, angiographic findings, and postoperative clinical and radiological outcomes were analyzed. Results are discussed in the light of a systematic literature review on reported true hybrid procedures for IA treatment. (3) Results: In total, 10 tHAs were performed. Of these, coiling and concomitant decompressive craniectomy or hematoma evacuation was performed on six occasions. In two patients, multiple IAs were treated with different modalities during the same procedure. In two patients, intraoperative conditions did not allow for complete IA clipping, and the remnant was coiled in the same session. The review of the literature revealed nine papers comprising 58 IAs treated with a tHA. (4) Conclusions: The need for a tHA for IA treatment is rare and limited to highly selective cases. In our experience, tHAs have been most valuable in an emergency setting concerning ruptured IAs. Furthermore, tHAs may also be considered in patients with multiple aneurysms in different vascular territories.

## 1. Introduction

Current treatment guidelines for management of intracranial aneurysms (IAs) essentially recommend conservative, endovascular treatment (EVT), or open microsurgical treatment procedures [1,2,3]. EVT has become increasingly popular in the last two decades and represents the preferred therapy option in many cases, particularly in IAs in the posterior circulation, in patients with confirmed vasospasms, and in elderly patients with significant medical comorbidities [4]. On the other hand, typical IAs with a wide neck or IAs located at the middle cerebral artery (MCA) bifurcation are preferentially treated with open microsurgery [5,6]. In patients with ruptured IAs and concomitant acute subdural hematoma (SDH) or large intraparenchymal hematoma, open surgery may be needed in order to evacuate the hematoma and treat the ruptured IA.

However, in a few select cases, neither EVT nor open microsurgical treatment alone may be sufficient for optimal IA treatment, and a combined approach combining endovascular and open surgical steps may be a valuable option. Previous studies reported numerous advantages of hybrid procedures for IA treatment, such as the option to perform direct intraoperative control angiography after clipping [7], to detect remnants [8], to perform intraoperative intravasal spasmolysis if needed [9,10], or for proximal control with a temporary balloon occlusion during clipping procedures [11,12,13,14]. However, the combination of endovascular and open surgical treatment combined in a single session using a true hybrid approach (tHA) in a hybrid operation room has only very rarely been described. We excluded enhancing procedures such as balloon occlusion and clipping of an aneurysm since the balloon occlusion does not have a permanent therapeutically effect.

In this study, we present our 12 years of experience with tHAs for IA treatment. Indications, procedural peculiarities, and patient outcome are reported. Furthermore, a systematic literature review was conducted to identify all relevant publications on tHAs for IA treatment and findings are critically discussed.

## 2. Materials and Methods

All patients treated with a tHA in our institution between January 2010 and January 2022 were included. “True hybrid” was defined as an intervention involving both open surgical and endovascular treatments in a single session in our hybrid OR. Examples include primary coiling plus a craniotomy or primary clipping incorporated with contemporary coiling (or potentially, a different flow reducing endovascular intervention). Combined endovascular and open surgical treatment for IA that did not take place in the same setting and session were excluded. Also, patients with intraoperative diagnostic digital subtraction angiography (DSA) or balloon occlusion but without additional endovascular treatment were not included. The study was approved by the local ethics committee (2020-02104) and individual patient consent was waived. Indication for hybrid surgery in elective cases was identified and performed based on clinical interdisciplinary consensus (neurosurgeons, interventional neuroradiologists, neurologists). In cases of emergency treatment, interdisciplinary ad hoc decisions were made only between neurosurgeons and interventional neuroradiologists, and clinical and radiological data were prospectively collected and retrospectively analyzed.

### 2.1. Clinical and Radiological Data

Patient characteristics (sex, age on admission, medical comorbidities, medication, smoking status, hypertension, drug abuse, and family history of aneurysms), clinical status on admission, Glasgow Coma Scale (GCS), modified Rankin Scale (mRS), neurological symptoms, aspects of the IA and/or subarachnoid hemorrhage (size, location, shape, calcification perianeurysmal environment, number of IAs, rupture status, symptomatic bleeding, new cranial nerve deficit, initial World Federation of Neurosurgery Score (WFNS) [15], initial modified Fisher scale [16], initial Hunt and Hess scale (H&H scale) [17], and number of pretreatments of the IA were recorded from the clinical information system. Surgical interventional data included total duration of intervention, duration of open surgery, duration of endovascular treatment, type of endovascular treatment, radiation dose, IA remnants, and potential clip adjustment maneuvers. Postsurgical neurological deficits, bleedings, and ischemia were also recorded. Postoperative functional outcome was measured via mRS, GOS, and GCS at discharge and the latest clinical follow-up. Radiological data included pre- and postoperative imaging (modality, date). Dates of clinical and radiological follow-ups were logged, and completeness of aneurysm occlusion, further treatments and interventions, and any complications correlating with the hybrid procedure were registered.

### 2.2. Hybrid Operation Settings

The setup and equipment of our hybrid operation room has been described previously [11]. Briefly, the surgical table (Alphamaquet 1150 (Maquet AG, Gossau, Switzerland)) is coupled with a uniplanar C-arm angiography system (Allura Xper FD20 (Philips, Amsterdam, The Netherlands)). Patients are placed on the carbon fiber table, and their heads are attached to radiolucent head holders (DORO systems (Pro Med Instruments (PMI), Freiburg, Germany)) or (Mayfield (Integra LifeSciences Corporation, Cincinnati, OH, USA)) and fixed with radiolucent pins.

### 2.3. Search of the Literature

**Figure 1:**
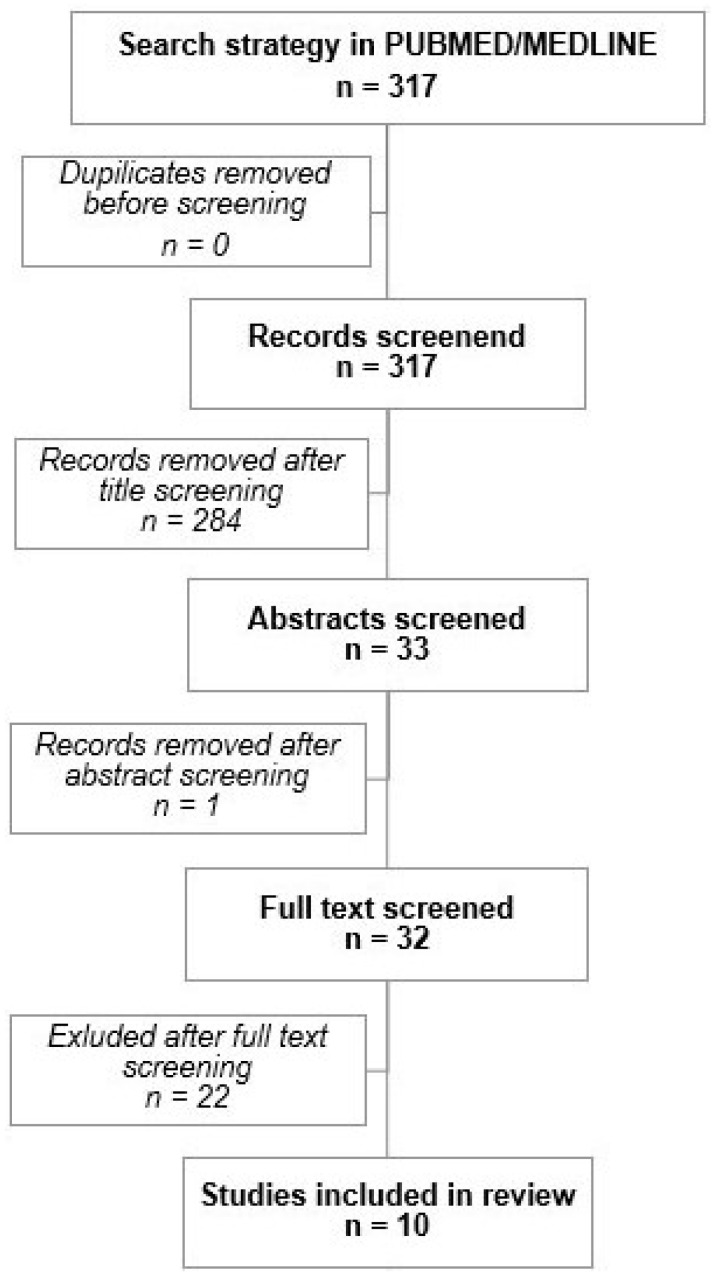
PRISMA flowchart for study selection. Of 279 studies identified by the search algorithm, 9 studies presented cases of true hybrid interventions and were included for review in the present study.

A systematic literature search was performed on PubMed/Medline for “aneurysm (brain OR cerebral) AND hybrid (surgery OR surgical OR treatment)”. All studies published until January 2023 were included. Duplicates were removed, and two authors from the current study individually screened titles, abstracts, and full texts for inclusion (Figure 1). All studies and case reports concerning tHAs for treatment of IAs were included. Non-clinical studies, those where procedures did not take place in a hybrid OR and where patients had to be transferred in between interventions were excluded [18]. Opinions, conference abstracts, and other non-original research were also excluded. Any discrepancy between the screening authors was discussed until consensus was reached. PRISMA guidelines were strictly applied [19].

## 3. Results

**Figure 2:**
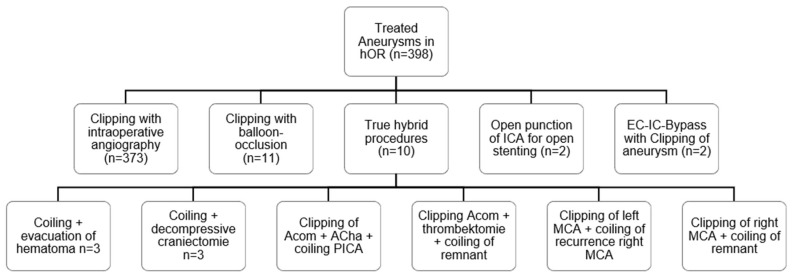
Overview of all hybrid procedures for IAs. Of 398 hybrid operations for treatment of IAs, only 10 operations were true hybrid procedures combining open and endovascular interventions.

In total, 398 combined surgical and endovascular IA hybrid operations (Figure 2) were identified during the study period. Of these, 10 (2.6%) were tHAs (Figure 2). Two cases were elective surgeries and eight were emergency surgeries after ruptured IAs. In nine cases, it was an ad hoc decision to perform a tHA and only one case was primarily electively planned as a tHA. Indications for a tHA included: hematoma evacuation in combination with aneurysm coiling (n = 3), decompressive craniectomy in combination with coiling (n = 3), clipping and coiling of multiple IAs in different vascular territories (n = 2), coiling of a remnant after incomplete clipping (n = 1), and emergency thrombectomy after intraoperative clot formation after clipping (n = 1). Details of all 10 hybrid procedures are given in Table 1.

### 3.1. Coiling and Open Surgical Hematoma Evacuation

Among three patients with coiling and hematoma evacuation, a 73-year-old female presented with a ruptured narrow-neck anterior communicating artery (Acom) aneurysm, resulting in a fulminant subarachnoidal, intracerebral, and intraventricular hemorrhage. Coiling and hematoma evacuation were performed successfully. A ventriculoperitoneal shunt was inserted eight days after initial surgery due to persistently high intra-cranial pressure (ICP). After 30 days, the patient was discharged to neuro-rehabilitation. Upon follow-up over 2.5 years, she had recovered to an mRS score of 2. Likewise, a 51-year-old male patient presented with a rupture of a right terminal internal carotid artery (ICA) aneurysm (C7). Coiling and evacuation of the temporoparietal hematoma was performed successfully. The patient consequently developed severe symptomatic vasospasms. The 3-month follow-up showed persistent left-sided hemiparesis. In the third case, a 64-year-old female patient presented with fulminant subarachnoid hemorrhage (SAH) and intracerebral frontotemporal hematoma. The diagnostic DSA performed in the hybrid operation room revealed a ruptured right narrow-neck MCA aneurysm, and a second, incidental aneurysm of the Acom. The ruptured aneurysm was immediately coiled, and to save time, the hematoma was surgically evacuated immediately. However, the ICP increased to >45 mmHg a few hours postoperatively, requiring emergency decompressive craniectomy. Despite a subsequent normalized ICP, a CT scan showed infarction in multiple vascular territories as well as generalized brain edema, and the patient died 13 days later.

### 3.2. Coiling and Decompressive Craniectomy

Coiling and a decompressive craniectomy was performed in three cases: two patients presented with ruptured aneurysms (one in the pericallosal artery and the other in the distal ICA) with concomitant subdural hematoma in both cases. In both cases, the aneurysms were successfully coiled, but because of extensive brain swelling, decompressive craniectomy and hematoma evacuation were performed. One of those patients recovered well with an mRS score of 2 at 6.5-year follow-up, while the other patient developed further complications and died one month later from pulmonary embolism. The third patient, a 44-year-old female, presented late (7 days) after initial symptoms. CT scan on admission showed fulminant SAH due to a ruptured left MCA aneurysm with severe intraventricular extension and obstructive hydrocephalus. Due to the already established severe vasospasms on the initial CTA, interdisciplinary consensus opted for tHA and coiling followed by immediate intra-arterial spasmolysis, which were successfully conducted. As the ICP remained increased until the end of the procedure, decompressive craniectomy was additionally performed. Despite continued spasmolytic therapy, the patient developed further severe vasospasms and died 13 days after admission.

### 3.3. Clipping of Multiple Aneurysms and Concomitant Coiling

**Figure 3:**
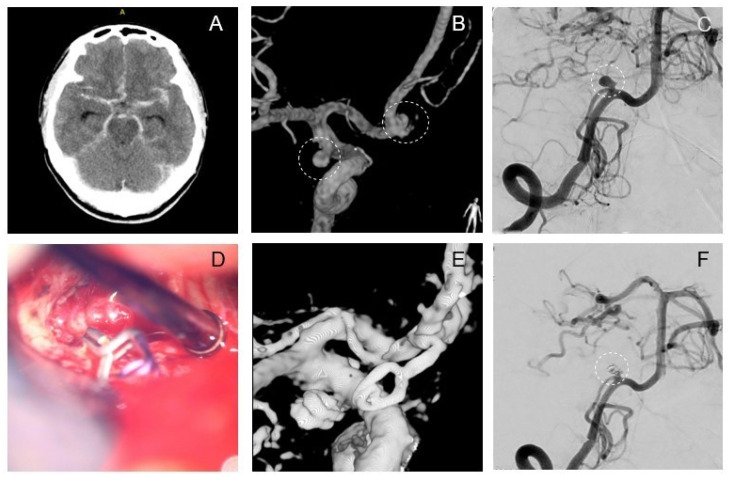
Exemplary case 1: Clipping of left Acom and AChA and coiling of right PICA. CT-Scan on admission shows aneurysmatic SAH (FISHER III, HH II, WFNS II) (**A**). Aneurysms of left Acom and the left anterior choroidal artery (AChA) are detected in DSA before surgery (**B**), in addition to a left PICA aneurysm (**C**). Intraoperative microscope view after successful clipping of the Acom aneurysm (**D**). Intraoperative 3D-angiography after clipping confirms complete obliteration of the aneurysms of the left Acom and anterior choroidal (**E**). The PICA aneurysm was then successfully treated by conventional coiling (**F**).

A 38-year-old female patient arrived at hospital with severe headaches and emesis following a car accident on the previous day. The initial CT scan with CTA revealed SAH (Fisher III, WFNS II, H&H II (Figure 3A)) and three IA: Acom, right anterior choroidal artery (ACha), and right posterior inferior cerebral artery (PICA) (Figure 3B,C). Because of suggestive blood distribution, an interdisciplinary decision was made to explore and clip both aneurysms of the anterior circulation, but intraoperatively neither the Acom nor the ACha aneurysm showed signs of rupture. Subsequently, the narrow-based PICA aneurysm was identified as the source of bleeding and completely coiled (Figure 3F) in the hybrid OR. Postoperatively, the patient presented with a GCS score of 14 and had not developed any neurological deficits. On the third postoperative day, she was transferred to a hospital in her home country and lost to further follow-up.

### 3.4. Clipping, Thrombectomy, and Coiling

**Figure 4:**
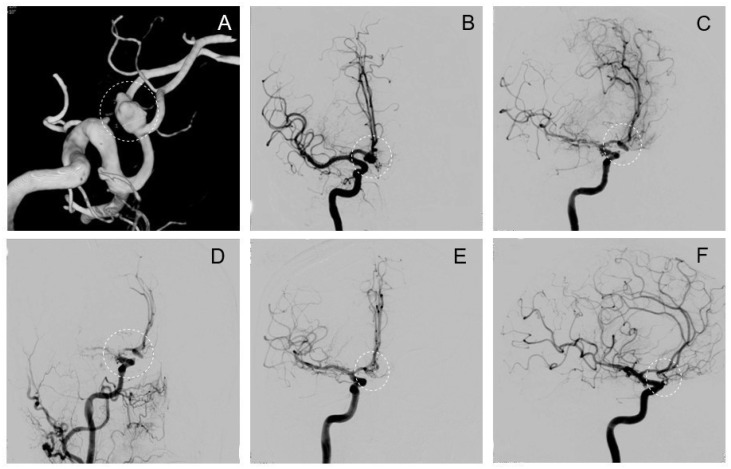
Exemplary case 2: Clipping, thrombectomy, and remnant coiling. A complex-shaped IA of the Acom complex in 3D (**A**) and frontal 2D (**B**) view. Intraoperative control angiography reveals a remnant (circle) after clipping ((**C**), oblique projection). After a few minutes, a thrombus formed in the MCA-M1 segment (probably due to the previous manipulation on the vessel) (**D**). Endovascular thrombectomy was successfully performed and the remnant aneurysm immediately coiled thereafter (**E**,**F**).

Initial interdisciplinary consensus suggested primarily EVT in the angiography suite in a 52-year-old female patient who presented with a ruptured Acom aneurysm (Figure 4A–C). However, positioning a framing coil in this broad-based aneurysm was technically challenging and the procedure was stopped prematurely. One day later, the patient was admitted to the hybrid OR and craniotomy was then performed followed by microsurgical clipping, which proved difficult due to substantial brain swelling. Intraoperative control DSA revealed a substantial aneurysm remnant (Figure 4D), but more alarmingly, also an occlusive thrombus in the MCA-M1 segment, including M1–M2 bifurcation (Figure 4E). Immediate endovascular thrombectomy was successfully performed. Because of the brain swelling, interdisciplinary decision to coil the remnant aneurysm was made (Figure 4F). Postoperatively, the patient developed a transient paresis of the left arm. The further course included therapy for symptomatic vasospasms. At discharge, the patient presented with a GCS score of 15 and distal M3 paresis of the right arm that further recovered to M5 at 3-month follow-up.

### 3.5. Clipping of the Right MCA Followed by Remnant Coiling

**Figure 5:**
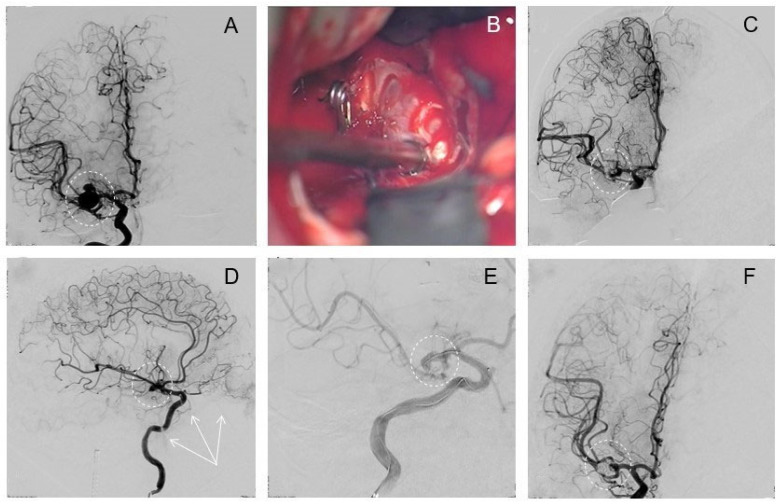
Exemplary case 3: Clipping followed by remnant coiling. Aneurysm of the right MCA before treatment (**A**). Intraoperative situs after clipping (**B**). Intraoperative control DSA in frontal (**C**) and lateral projection (**D**) reveals a persisting remnant on the neck of the MCA aneurysm after clipping (circle). Note the craniotomy site is still open (fishhooks holding myocutaneous flap, arrows). After intraoperative coiling of the remnant (**E**), the aneurysm is completely obliterated (**F**).

**Figure 6:**
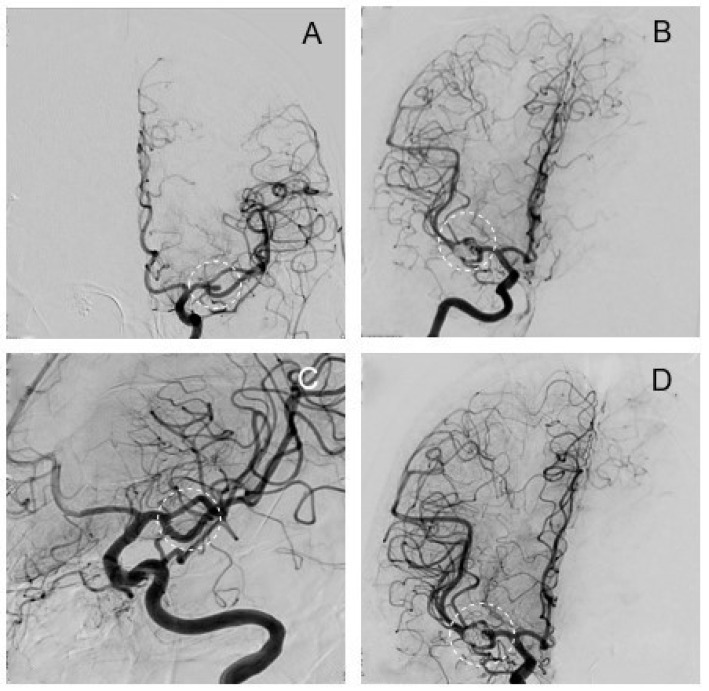
Exemplary case 4: Clipping of a left MCA and re-coiling of a right MCA aneurysm. Left MCA aneurysm before surgery (**A**). Right recurrent MCA aneurysm before re-coiling (**B**). Intraoperative iDSA of left MCA after clipping shows complete aneurysm obliteration (**C**). The right MCA aneurysm after re-coiling is also completely obliterated (**D**).

A 51-year-old left-handed female patient was electively treated for an incidental right MCA aneurysm and diagnosed with a smaller contralateral left MCA mirror aneurysm. (Figure 5A). Dissection of the aneurysm was challenging due to distinctive adhesions. Intraoperative control DSA showed a remnant after clipping (Figure 5C,D), but given the patient’s right hemispheric dominance and adhesions with the M2 segment at the aneurysm fundus, repositioning of the clip and further manipulation was considered too risky. Instead, the operation was passed on to the neurointerventionists, who coiled the remnant in the same session in our hybrid OR. (Figure 5E). Postoperatively, the patient developed a transient aphasia and a mild paresis of the left arm, which had recovered to mild fine motor skill impairment, but showed no significant paresis after rehabilitation, at 6-month follow-up. Radiological follow-up at 10 months, however, showed a recurrence of the treated IA (Figure 6A).

### 3.6. Clipping of Left MCA and Re-Coiling of Right MCA (Elective)

Ten months after the first operation (detailed description above), the now 52-year-old female patient was readmitted for elective clipping of the incidentally found left-sided mirror MCA aneurysm and consecutive re-coiling of the recurrent, previously clipped and coiled right MCA aneurysm. The decision to perform both procedures in a single session was made, and so the patient’s strong wish to do it within the same hospitalization was respected. Clipping of the left MCA was performed and intraoperative control angiography showed complete occlusion (Figure 6C). Coiling of the previously treated right MCA aneurysm was also successful (Figure 6D). She was discharged shortly after without any new deficits. Radiological follow-up after 4 years showed no recurrence on either side.

### 3.7. Literature Review

The search revealed 10 publications that reported a tHA for IA treatment (Table 2). More specifically, hybrid procedures included several cases with coiling and evacuation of intracranial hematoma [20,21], partial embolization and clipping of remnant [22], or initial clipping and coiling of the remnant IA [23]. Hybrid combinations also comprised IC–EC bypass (STA–MCA, CCA–MCA and OA–PICA, OA–SCA) in combination with endovascular trapping [22,24,25,26] or endovascular coil embolization [20,26,27].

## 4. Discussion

This series identified 10 cases treated with a tHA out of nearly 400 surgeries performed on IAs over a period of 12 years. Of these, six procedures combined endovascular IA occlusion with a basic neurosurgical (non-vascular) operation, such as intracranial hematoma evacuation or decompressive hemicraniectomy. In two cases, the tHA enabled treatment of multiple aneurysms in different vascular territories or in contralateral hemispheres within one single procedure. Lastly, in two cases, one modality was not sufficient, but complete IA obliteration was achieved with a combination of clipping and coiling in the same operation. Conceptually similar, a few cases of an extra-intracranial bypass in combination with endovascular tapping are described in the literature.

### 4.1. Coiling and Craniotomy/Decompressive Craniectomy

In some cases, with ruptured IAs and large intracranial hematoma, coiling may still be the preferred modality to approach the IA, whereas a hematoma requires craniotomy and evacuation. This indication has been reported by several other groups [20,21,22]. Advantages include the avoidance of a deep Sylvian preparation in a difficult situs of swollen brain and the security of an already protected aneurysm in the depth of the hematoma during its evacuation and thus no requirement for a vascularly trained neurosurgeon. Also, the need for fewer resources and the time saved so the patient does not have to be transported from the angiography suite and the OR is one reason to perform both interventions in the same session in the same OR suite. On the other hand, decompressive hemicraniectomy is less usual when dealing with SAH. There are no previous reports on coiling in combination with decompressive hemicraniectomy. Our series contains three cases, all with delayed presentation after the rupture of the IA and with uncontrollable high ICP despite maximal conservative therapy and invasive ICP management. Consequently, a decompressive hemicraniectomy was performed in combination with concomitant coiling. In two cases, the IAs were approached endovascularly due to the localization, size, and morphology (distal ICA, DACA), and in one case due to severe vasospasms requiring endovascular spasmolysis therapy [30]. Whereas one patient survived the acute phase and eventually revealed a good clinical outcome (mRS 2) at over 6.5 years, the other two patients died on postoperative days 13 and 36, respectively. These outcomes indicate that coiling with decompression is a procedure that may only be considered as a last resort in critically ill patients, and is associated with poor overall outcomes.

### 4.2. Multimodal Treatment of Multiple Aneurysms in One Session

Our series includes two patients in which multiple aneurysms in different vascular territories were treated with different modalities. Combination of clipping and coiling of multiple aneurysms or aneurysm remnants has been previously reported [20,23,29]. Although there is no absolute need to approach multiple IAs in one session, in both cases there was a clear benefit in comparison to a staged approach with two separate interventions. In the first case, the patient presented with acute SAH and multiple aneurysms (Acom, ACha, and PICA). Blood distribution in the basal cisterns was indicative of the Acom as the most likely source of the bleeding, which was also the largest IA of the three. Intraoperative inspection, however, showed that neither the Acom aneurysm nor the ACha aneurysm had ruptured and was the source of the bleeding. Approaching the PICA was not possible through the same craniotomy, but this last IA could immediately be treated endovascularly without further delay, reducing the potential for a rebleed.

The second case was a patient with MCA mirror aneurysms of which the right side had previously been clipped and coiled, with coil compaction and recurrent perfusion at the aneurysm neck over 6 months with need for re-coiling. As treatment was also indicated for the contralateral IA, it was her strong preference to undergo treatment for both at the same time with only one hospitalization and one narcosis. From a medical point of view, a staged approach would have been reasonable too, but fulfilling her wish for a tHA increased the patient’s comfort and reduced overall costs.

### 4.3. Multimodal Treatment of a Single IA or IA in the Same Vascular Territory

In two cases, a single-modality treatment was not sufficient to treat the IA completely or the initial modality selection proved insufficient when maintaining a reasonably low risk profile. On the other hand (in a patient with a ruptured Acom IA and where there were difficulties in placing a framing coil), clip positioning did not fully exclude the IA from perfusion, but made it more suitable for coiling and eventual complete thrombosis. In the literature, this multimodal approach is most frequently reported as combined extra-intracranial bypass surgery with endovascular trapping or IA embolization [20,22,24,25,26,27,28].

### 4.4. Complications

There were no complications, related or unrelated, to the tHA in this series. By contrast, the tHA may have prevented potentially fatal complications by avoiding unnecessary risks, i.e., switching from primary coiling to clipping when it was not possible to anchor the first coil or leaving a remnant for coiling after primary clipping instead of performing a risky clip adjustment maneuver. In addition, the rigorous pre-interventional discussion of every case with interdisciplinary consent regarding treatment indication and modality (mandatory for all hybrid operations) may help avoid suboptimal treatments and complications. However, it is important to note that—particularly in a setting of ruptured intracranial aneurysms—patients who qualify for a tHA are often in a serious condition. A simpler procedure may not have been sufficient (i.e., coiling without hematoma evacuation) or not even possible (i.e., clipping of a spastic vessel in the depth of a swollen brain), and a tHA may be seen as a last-chance rescue maneuver. This could explain why three patients died within a few days postop. None of them, however, experienced procedure-related complications. Furthermore, a tHA facilitates immediate reaction upon detection of ultra-early periinterventional vasospasm and may therefore be particularly beneficial in severely affected patients [10,30]. Lastly, with regard to radiation exposure, no additional endovascular procedures were performed. It is a question of timing if an endovascular and an open surgical procedure should be performed as a tHA in a single session, or alternatively as a staged approach in two separate interventions.

### 4.5. Strengths and Limitations

Indications for a tHA in IA surgery are extremely rare. The present series is among the largest published and summarizes all the cases in the literature to date. However, overall case numbers remain low and indication for hybrid treatment remains highly selective and is mostly an ad hoc decision in emergency situations. Also to be considered should be the costs of a hybrid OR and its equipment. It is more expensive to build a fully equipped hybrid OR than a regular, non-hybrid OR. Furthermore, in the short term, it is also more expensive to keep a hybrid OR running (i.e., personal, anesthesia time, etc.). Given the highly select indications for a true hybrid OR, this may probably not justify demanding a hybrid OR as a standard of care. However, if a hybrid OR is available, it may be beneficial in highly select patients.

## 5. Conclusions

The necessity for a true hybrid IA treatment is extremely rare. However, if a hybrid operation room is available, a tHA may be beneficial in highly selective cases in emergency situations. These cases include patients with ruptured aneurysms who undergo coiling and require emergency decompressive craniectomy or hematoma evacuation. Furthermore, a tHA might also be considered in individuals with multiple aneurysms in different vascular territories, or if a single treatment modality fails to achieve complete IA obliteration.

## Figures and Tables

**Table 1:** Summary of all true hybrid cases treated between November 2010 and January 2022.

#	Age	Sex	Site of Aneurysm	SAH	Peculiarity	True Hybrid Procedure	Follow-Up	mRS at Follow-Up
1	73	Female	Acom	Modified Fisher IV	Intraventricular and subdural hematoma	Coiling and hematoma evacuation	3 years	2
2	51	Male	Right distal ICA	Modified Fisher IV, WFNS IV, H&H III	Intraventricular and intracerebral hematoma	Coiling and hematoma evacuation	106 days (then lost to follow-up)	4
3	64	Female	Right MCA	Modified Fisher IV, WFNS V, H&H V	Subdural and intracerebral	Coiling and hematoma evacuation	13 days	6
4	44	Female	Left MCA	Modified Fisher IV, WFNS V, H&H IV	ICP 45 mmHg, intraventricular and intracerebral hematoma	Coiling, hematoma evacuation, and decompressive craniectomy	13 days	6
5	45	Female	Right pericallosal artery	Modified Fisher IV, WFNS I, H&H I	ICP 45 mmHg, subdural hematoma	Coiling and decompressive craniectomy	6.5 years	2
6	59	Male	Right distal ICA	Modified Fisher IV, WFNS II, H&H III	ICP 40 mmHg, subdural hematoma	Coiling and decompressive craniectomy	37 days	6
7	52	Female	Acom	Modified Fisher III, WFNS III, H&H III	Thrombus after clipping and remnant of aneurysm	Clipping, thrombectomy and coiling	10 years	1
8	38	Female	Acom, right ACha, right PICA	Modified Fisher III, WFNS II, H&H II	Multiple aneurysms in different vascular territories and unclear source of bleeding	Clipping of multiple aneurysms and concomitant coiling	Lost to follow-up	unknown
9	51	Female	Right MCA	none	Remnant after clipping with distinctive adhesions	Clipping and coiling of remnant	4 years, 9 months	1
10	52	Female	Left and right MCA	none	Recurrence of previously treated aneurysm and second aneurysm of opposite side	Coiling of recurrence on the right and clipping of left MCA aneurysm	4 years	1

**Table 2:** Studies reporting tHA for treatment of IAs.

Author, Year	Study Type	Number of Treated Aneurysms	Indications	Complications	Peculiarity	Study Conclusion
Iihara et al., 2013 [24]	Retrospective review	n = 2	STA–MCA bypass and endovascular trapping	none		Integration of a hybrid OR enables combined endovascular and surgical procedures for complex neurovascular and brachiocephalic lesions in a 1-stage treatment.
Murayama et al., 2013 [20]	Retrospective review	n = 9	STA–MCA bypass and embolization; coiling and hematoma evacuation; coiling and clipping	n = 3 Bypass occlusion, transient ischemic symptoms		Combined treatment is a superior option to avoid bypass occlusion. In cases of brain swelling after hemorrhage, coiling is safer than clipping.
Mori et al., 2016 [21]	Case report	n = 2	Coiling and hematoma evacuation	none	Endoscopic evacuation of hematoma	Short transition time could be favorable for outcome.
Kawamura et al., 2017 [25]	Case report	n = 1	CCA–MCA bypass with radial artery graft and endovascular trapping	none	Aberrant ICA with pseudoaneurysm	True hybrid treatment is beneficial for treatment of aberrant ICA aneurysms and pseudoaneurysms.
Xin et al., 2018 [23]	Case report	n = 1	Clipping and coiling of remnant	none	Complete IA occlusion after the procedure	A true hybrid approach could offer an alternative for intraoperative IA remnants.
Fukuda et al., 2019 [27]	Case report	n = 1	STA–MCA bypass with distal clipping and coil embolization	none	Traumatic cerebral aneurysm	Procedure only possible in hybrid operating room.
Jeon et al., 2019 [22]	Retrospective review	n = 15	OA–PICA bypass and endovascular trapping; partial embolization and clipping with/without ICH evacuation	none		Combined endovascular and surgical approach would provide new strategies for complex cerebrovascular diseases.
Wang et al., 2020 [28]	Retrospective review	n = 22	STA–MCA bypass with coil embolization	none		STA–MCA bypass in combination with coiling is a good option for complex cerebral aneurysms where surgical clipping or endovascular embolization is not a good solution.
Rotim et al., 2021 [29]	Case series	n = 5	Coiling and clipping of multiple aneurysms	none	Multiple aneurysms in different vascular territory treated in one session	True hybrid approach may be used when a single modality is insufficient to bring satisfactory results
Ogura et al., 2023 [26]	Case report	n = 1	OA–SCA bypass with STA graft and endovascular trapping and embolization	none		Combined surgical and endovascular approach is a treatment option for distal SCA aneurysms when there are concerns regarding ischemia following parent artery occlusion
Current study 2023	Retrospective review	n = 10	Coiling with need for decompressive craniectomy, coiling and hematoma evacuation, clipping and coiling of multiple aneurysms or coiling of remnant after clipping	none		True hybrid approach may be beneficial in highly selective cases, for instance, ruptured IAs that undergo coiling with need of craniotomy for hematoma evacuation/decompression or patients with multiple aneurysms in different vascular territories

## Data Availability

The data that support the findings of this study are available from the corresponding author upon reasonable request. The data are not publicly available due to privacy and ethical restrictions.
